# Expression of Concern: Paroxetine treatment in an animal model of depression improves sperm quality

**DOI:** 10.1371/journal.pone.0323480

**Published:** 2025-04-24

**Authors:** The PLOS One Editors

After publication of this article [1], concerns were raised about results presented in Figs 3-7. Specifically:

- The following panels appear similar to each other:
  ◦ Fig 3 B1 and Fig 4A
  ◦ Fig 3 C1 and Fig 4D
  ◦ Fig 3 E1 and Fig 4B
- The significance values in Figs 3-7 are letters that are not individually defined in the legends.

Corresponding author MHNE stated that the positioning and labelling of the panels in Fig 4 is incorrect. An updated version of Fig 4 is provided in S5 File. Corresponding author MHNE furthermore stated that the above-listed similar panel pairs in Figs 3 and 4 are not duplicates, but appear highly similar because they are from consecutive serial sections. The underlying images for some of the panels in Figs 3 and 4 are provided here in S2 File. Two members of the *PLOS One* Editorial Board reviewed the images in Figs 3 and 4, and while it was noted that use of consecutive sections that often display similar features is a standard histological practice, one member of the Editorial Board advised that this explanation does not resolve the concerns as continuous images should show differences.

Corresponding author MHNE provided updated versions of Figs 3 and 4 (S5-S6 Files) which contain an additional column in the integrated tables for p-values and updated values for mean and SEM. The corresponding author MHNE stated that a number of factors contributed to the different mean and SEM values reported in the updated figures, as follows:

- The data were reanalyzed based on the raw data; previous SPSS outputs are no longer available. The reanalysis resulted in different values due to changes in rounding.
- In some rows of the originally published tables, SD was reported in error.
- In some instances a decimal point was incorrectly inserted instead of a zero during the original analysis.
- In the originally published Fig 3 table, for Leydig Control, the mean was incorrectly reported as 2.21, rather than 2.12 because of a typing error.
- Some letters indicating the difference between the means of the experimental groups in the originally published tables in Figs 3 and 4 were entered incorrectly.

A statistical reviewer assessed the updated versions of Figs 3 and 4 (S5-S6 Files) and noted the following concerns:

- There appears to be selective reporting of p-values where variable numbers of p-values are reported meaning that non-significant p-values appear to be omitted.
- There does not appear to be adjustment for multiple testing, and as such some results could be chance findings.
- The number of decimal places reported in the revised tables is not consistent.
- The raw data underlying the initial erroneous analyses and full details of incorrectly inputted values were not provided, so it is not possible to independently assess the explanations for the changes.

A member of the *PLOS One* Editorial Board reviewed the updated versions of Figs 3 and 4 and noted that if the assays in question were removed from consideration, the overarching conclusion of the study—that depression negatively affects sperm quality, and paroxetine restores some parameters—remains supported by other findings in the article [1].

Corresponding author MHNE noted that there is an error in the Results section ‘Sperm parameters and sperm functional tests in the different animal Groups’ where the reference to Figs 3B-5A should be Figs 5A-5B and the reference to Figs 3F-5C should be Figs 5C-5F.

In light of the statistical concerns with Figs 3-7 that have not been fully resolved, and the errors in reporting image and quantitative data detailed above, the *PLOS One* Editors issue this Expression of Concern.

## Supporting Information

S1 File Underlying quantitative data for Figs 3 and 4. (XLSX)

S2 File Underlying images for Figs 3B, 3C, 3E, 3B1, 3C1 and the corrected Figs 4B and C. (JPG)

S3 File Supporting file for table in Fig 4. (DOCX)

S4 File Supporting file for table in Fig 3. (DOCX)

S5 File The updated version of Fig 4. Fig 4. Immunohistochemical staining of TUNEL positive cells in testes cross-sections. Comparison of mean percentage of TUNEL-positive cells (spermatogonia, spermatocytes, spermatids, Leydig, and Sertoli cells) within groups (N = 4). Mean ± SEM.
(TIF)

S6 File The updated version of Fig 3. Fig 3. Immunohistochemical staining of CASPASE-3 in testes cross-sections. Comparison of mean percentage of Caspase-3 -positive cells in germ cells (spermatogonia, spermatocytes, spermatids, Leydig, and Sertoli cells) within groups (N = 4). Mean ± SEM.
(TIF)
