# Supplementary figures and images for: Expression of Concern: Paroxetine treatment in an animal model of depression improves sperm quality
Source: PLoS One. 2025 Apr 24;20(4):e0323480. doi: 10.1371/journal.pone.0323480 (PMC12021233; doi:10.1371/journal.pone.0323480)

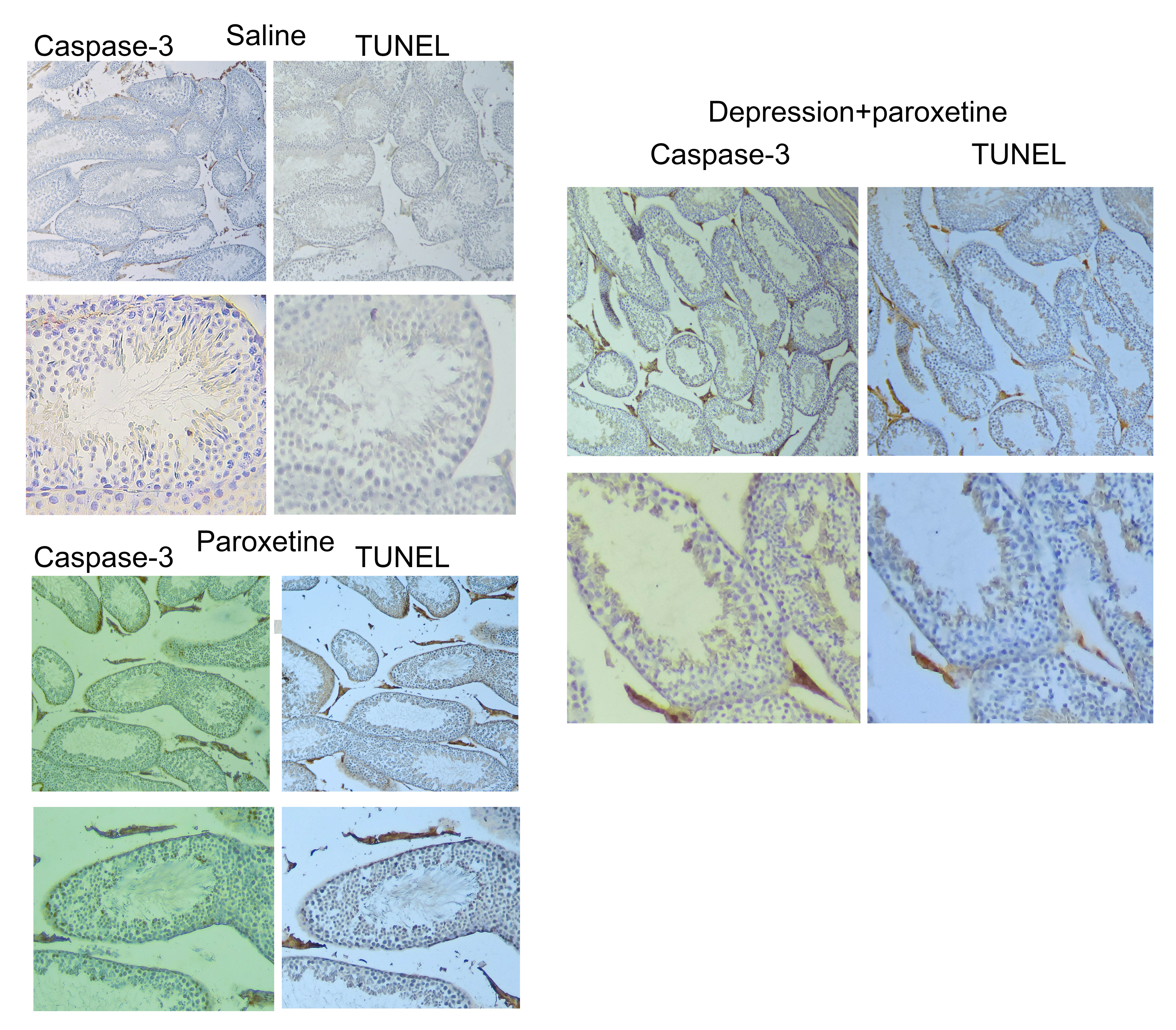

Supplement: S2 File — (JPG) [file pone.0323480.s002.jpg]

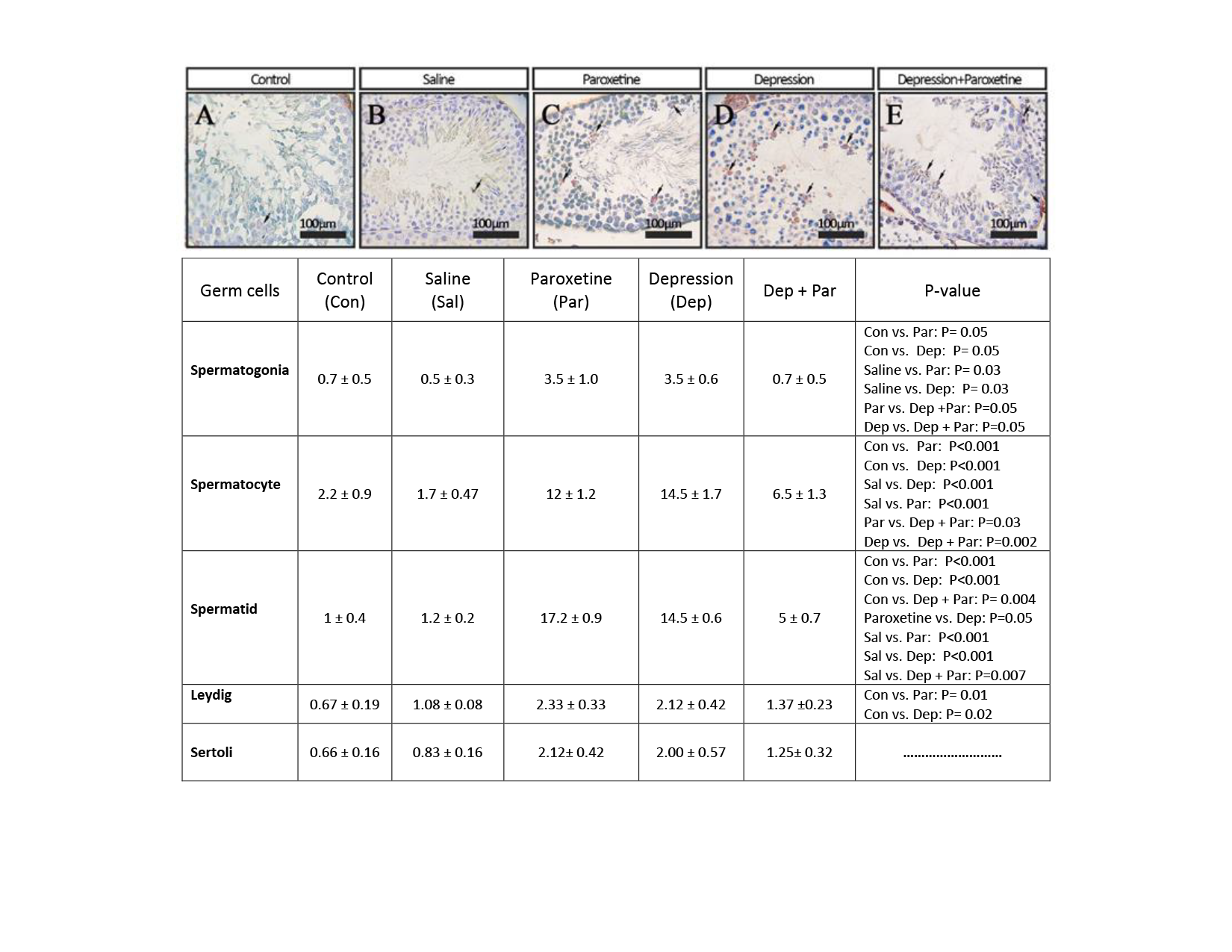

Supplement: S5 File — Fig 4. Immunohistochemical staining of TUNEL positive cells in testes cross-sections. Comparison of mean percentage of TUNEL-positive cells (spermatogonia, spermatocytes, spermatids, Leydig, and Sertoli cells) within groups (N = 4). Mean ± SEM. (TIF) [file pone.0323480.s005.tif]

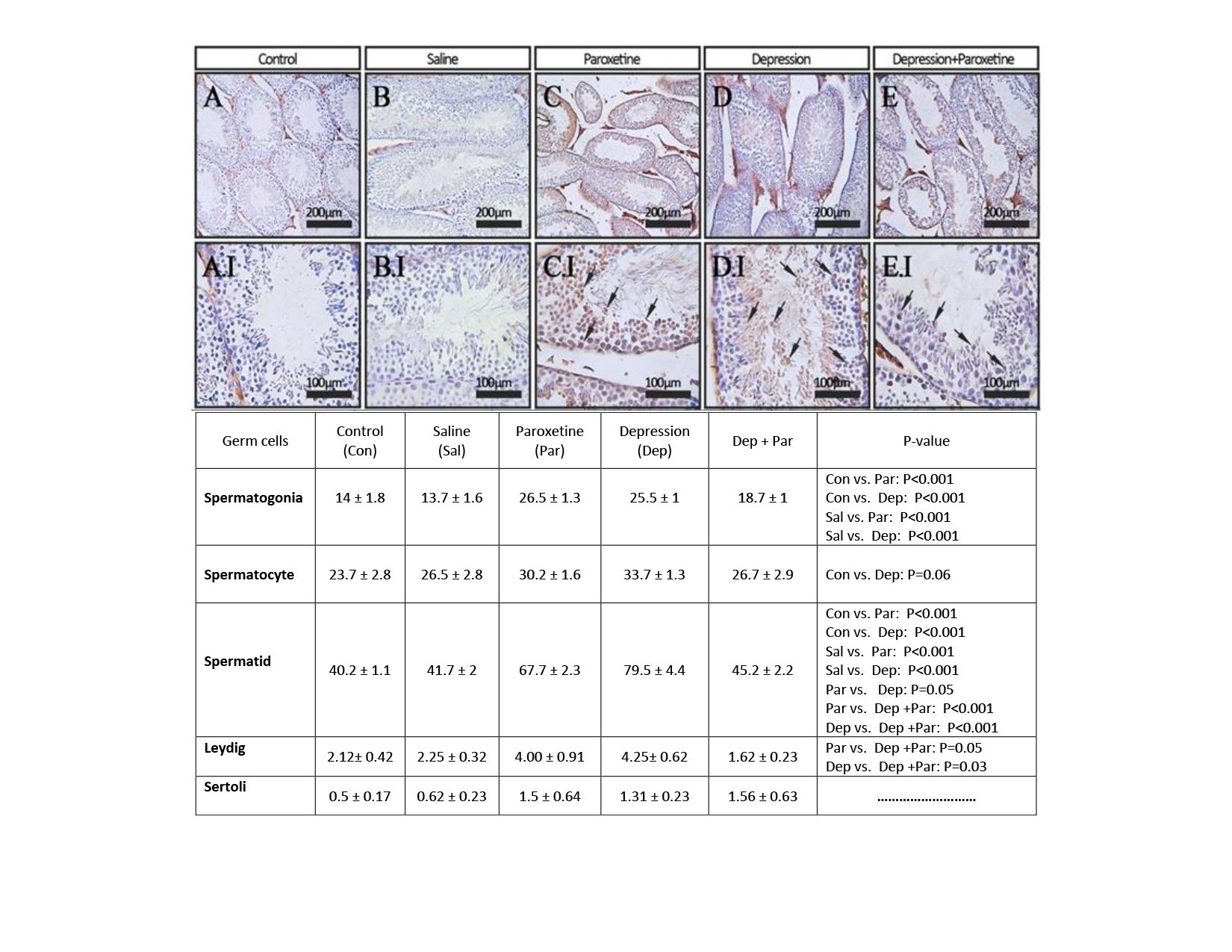

Supplement: S6 File — Fig 3. Immunohistochemical staining of CASPASE-3 in testes cross-sections. Comparison of mean percentage of Caspase-3 -positive cells in germ cells (spermatogonia, spermatocytes, spermatids, Leydig, and Sertoli cells) within groups (N = 4). Mean ± SEM. (TIF) [file pone.0323480.s006.tif]
