# Supplementary material for: Expression of Concern: Paroxetine treatment in an animal model of depression improves sperm quality
Source: PLoS One. 2025 Apr 24;20(4):e0323480. doi: 10.1371/journal.pone.0323480 (PMC12021233; doi:10.1371/journal.pone.0323480)

**Details of Table 4**

| Germ cells | Control | Saline | Paroxetine | Depression | Depression  +  Par | P-value |
| --- | --- | --- | --- | --- | --- | --- |
| **Spermatogonia**  Mean ± SEM  Mean ± SD | 0.7 ± 0.5  0.7 ± 0.9 | 0.5 ± 0.3  0.5 ± 0.6 | 3.5 ± 1.0  3.5 ± 2.0 | 3.5 ± 0.6  3.5 ± 1.3 | 0.75 ± 0.5  0.75 ± 0.9 | Control vs. Paroxetine: P= 0.05  Control vs. Depression: P= 0.05  Saline vs. Paroxetine: P= 0.03  Saline vs. Depression: P= 0.03  Paroxetine vs. Depression+  Par: P=0.05  Depression vs. Depression+  Par: P=0.05 |
| **Spermatocyte**  Mean ± SEM  Mean ± SD | 2.25 ± 0.9  2.25 ± 1.9 | 1.75 ± 0.5  1.75 ± 0.9 | 12 ± 1.2  12 ± 2.5 | 14.5 ± 1.7  14.5 ± 3.4 | 6.5 ± 1.3  6.5 ± 2.6 | Control vs. Paroxetine: P<0.001  Control vs. Depression: P<0.001  Saline vs. Depression: P<0.001  Saline vs. Paroxetine: P<0.001  Paroxetine vs. Depression+  Par: P=0.03  Depression vs. Depression+  Par: P=0.002 |
| **Spermatid**  Mean ± SEM  Mean ± SD | 1 ± 0.4  1 ± 0.8 | 1.25 ± 0.2  1.25 ± 0.5 | 17.25 ± 0.9  17.25 ± 1.9 | 14.5 ± 0.6  14.5 ±1.3 | 5 ± 0.7  5 ± 1.4 | Control vs. Paroxetine: P<0.001  Control vs. Depression: P<0.001  Control vs. Depression+  Par: P= 0.004  Paroxetine vs. Depression: P=0.05  Saline vs. Paroxetine: P<0.001  Saline vs. Depression: P<0.001  Saline vs. Depression+  Par: P=0.007 |
| **Leydig**  Mean ± SEM  Mean ± SD | 0.67 ± 0.2  0.67 ± 0.4 | 1.08 ± 0.08  1.08 ± 0.1 | 2.33 ± 0.4  2.33 ± 0.5 | 2.12 ± 0.4  2.12 ± 0.8 | 1.37 ±0.2  1.37 ± 0.47 | Control vs. Paroxetine: P= 0.01  Control vs. Depression: P= 0.02 |
| **Sertoli**  Mean ± SEM  Mean ± SD | 0.66 ± 0.16  0.66 ± 0.28 | 0.83 ± 0.1  0.83 ± 0.3 | 2.12± 0.4  2.12 ± 0.8 | 2.00 ± 0.6  2.00 ± 1.0 | 1.25± 0.3  1.25 ± 0.6 | **………………………** |

Figure 4: Immunohistochemical staining of TUNEL positive cells in testes cross-sections. Comparison of mean percentage of TUNEL-positive cells (spermatogonia, spermatocytes, spermatids, Leydig, and Sertoli cells) within groups.


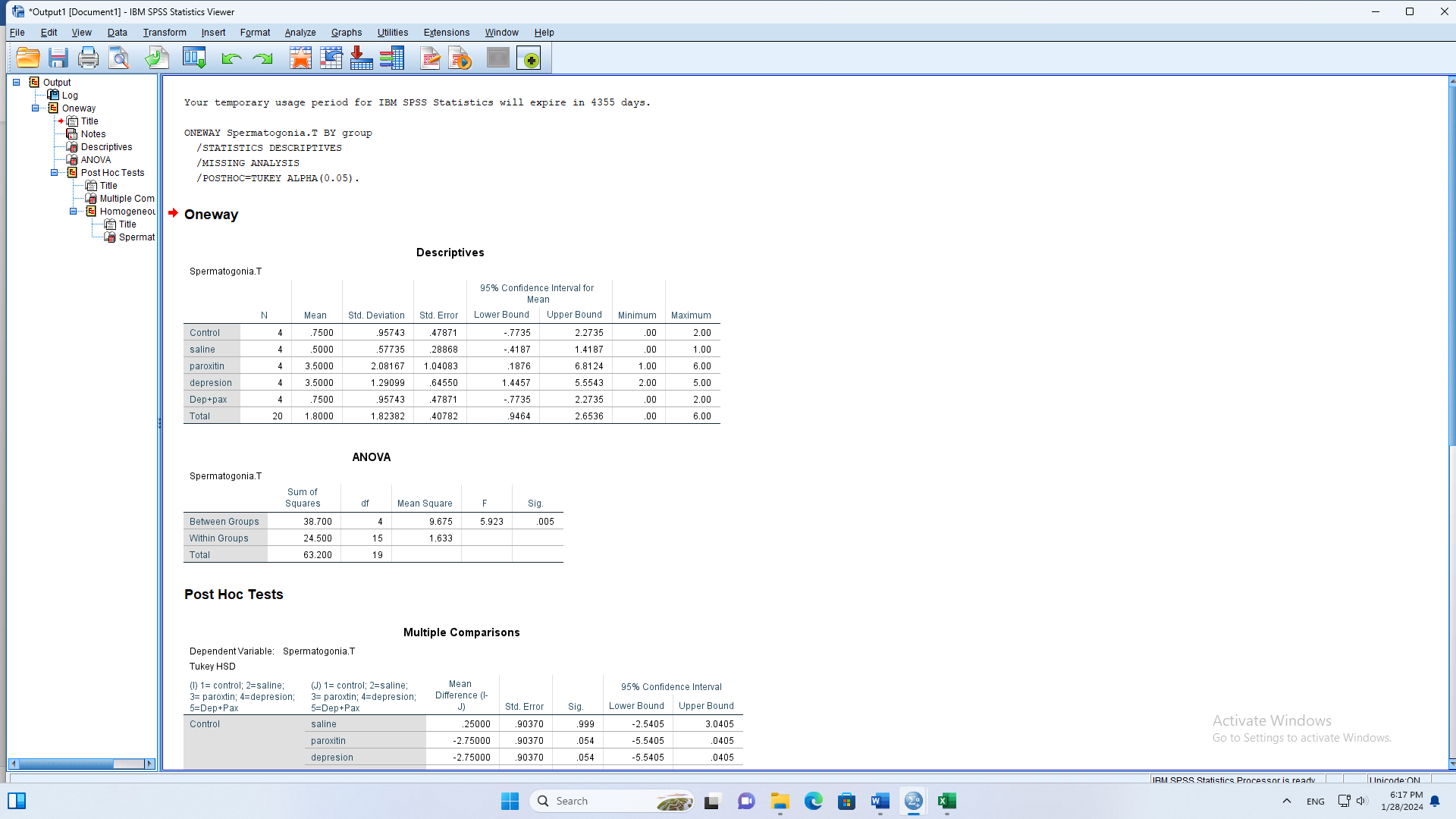

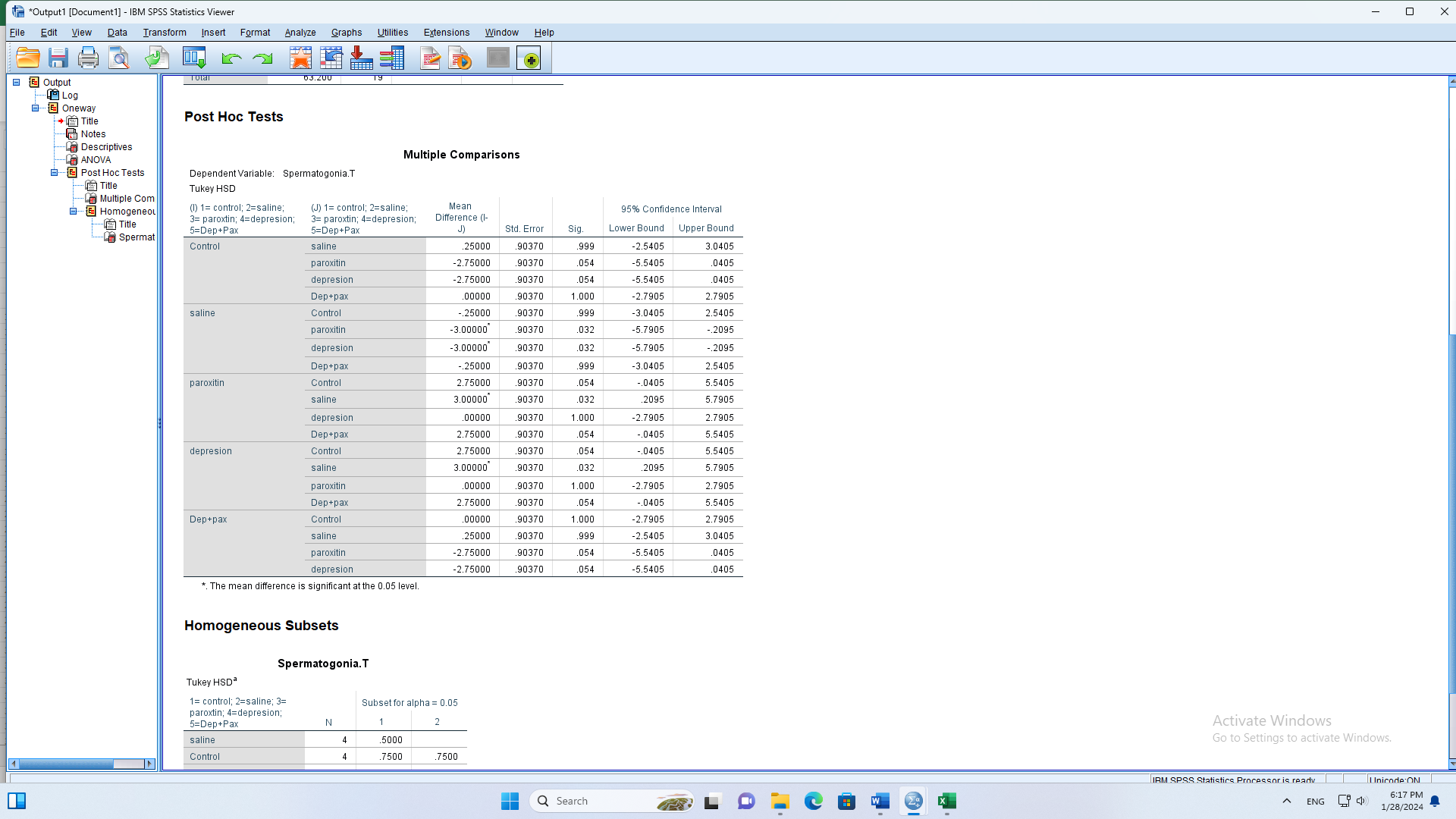


**…………………………………………………………………………………………………….**


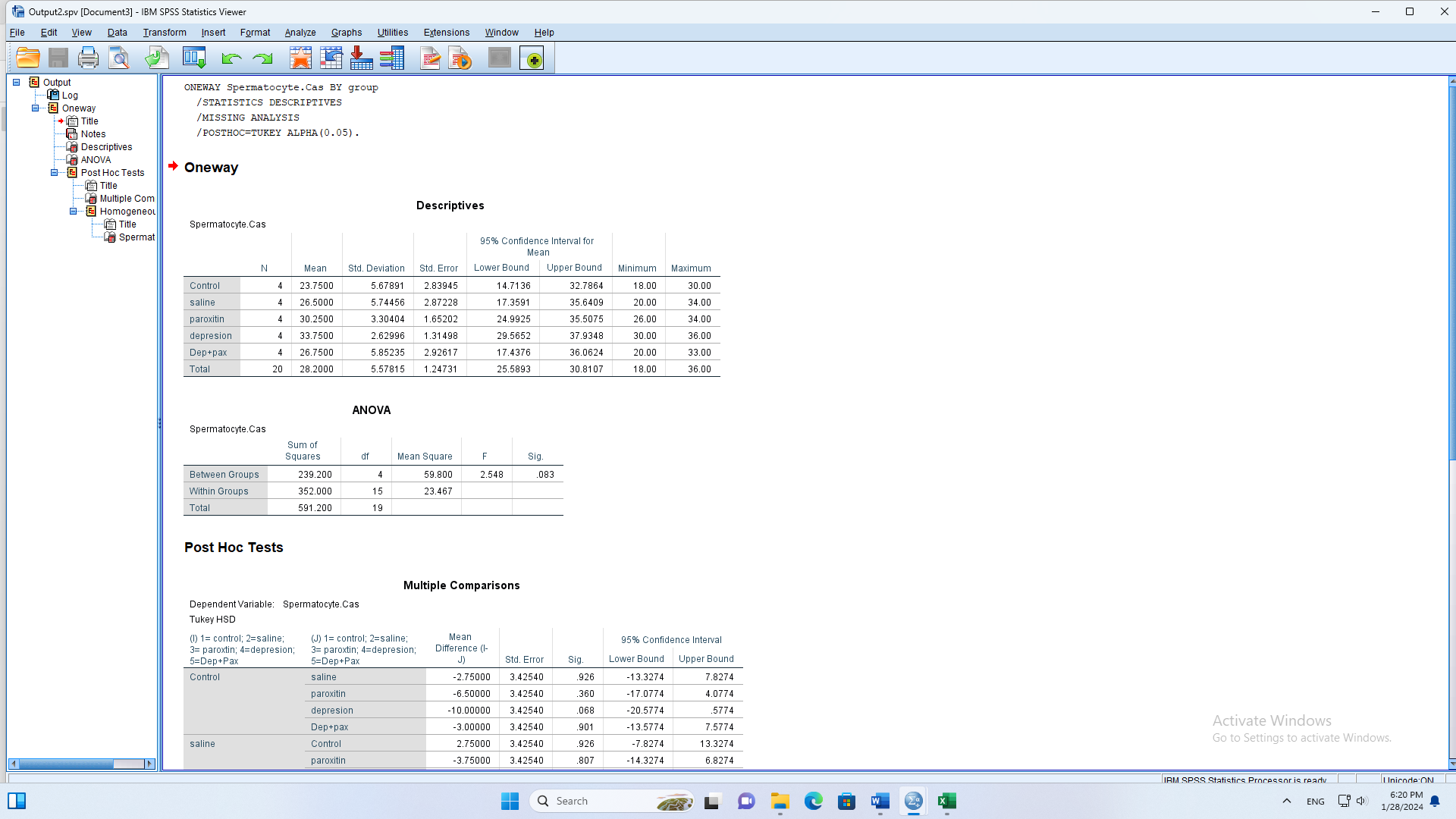

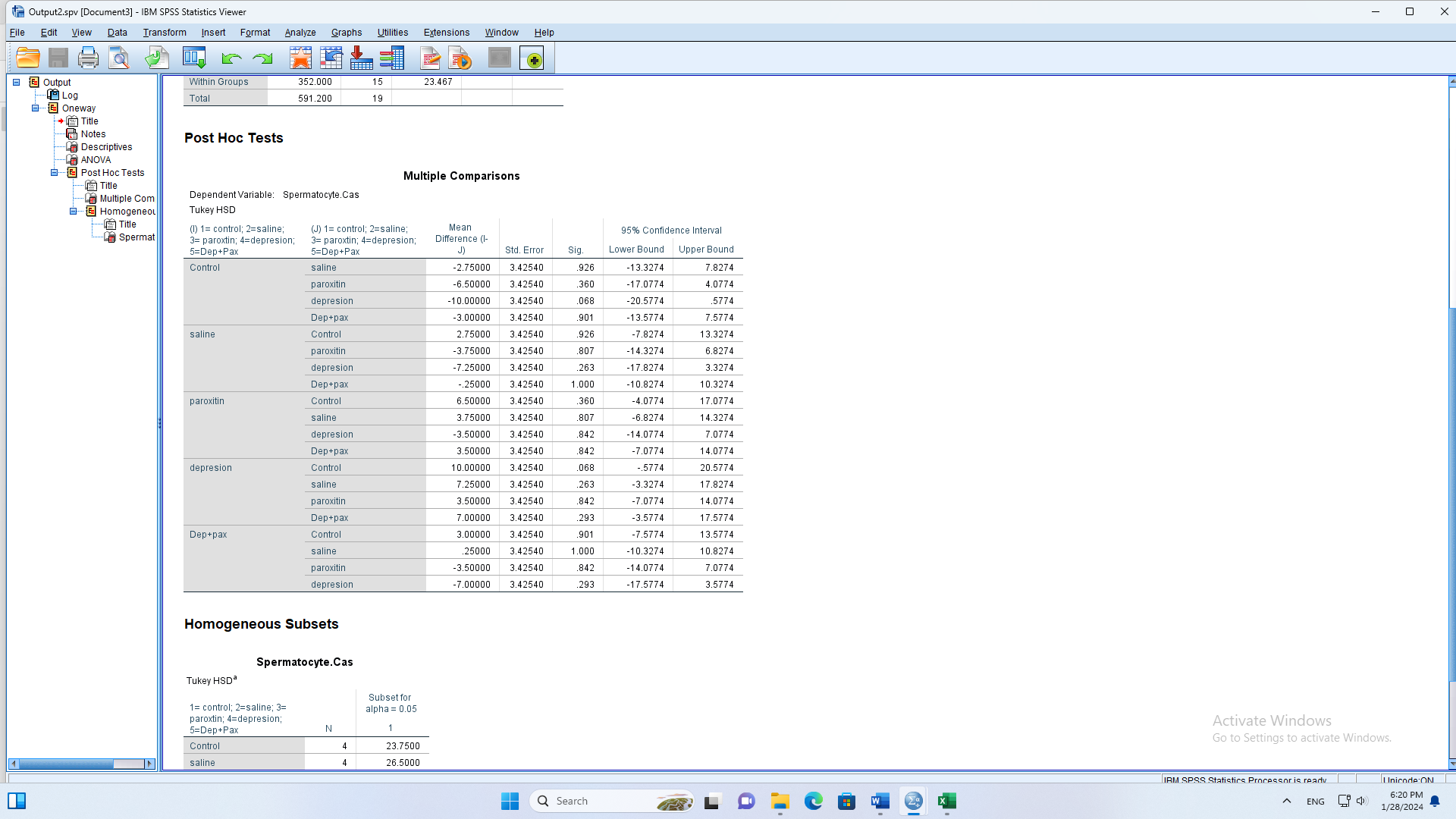


**…………………………………………………………………………………………………….**


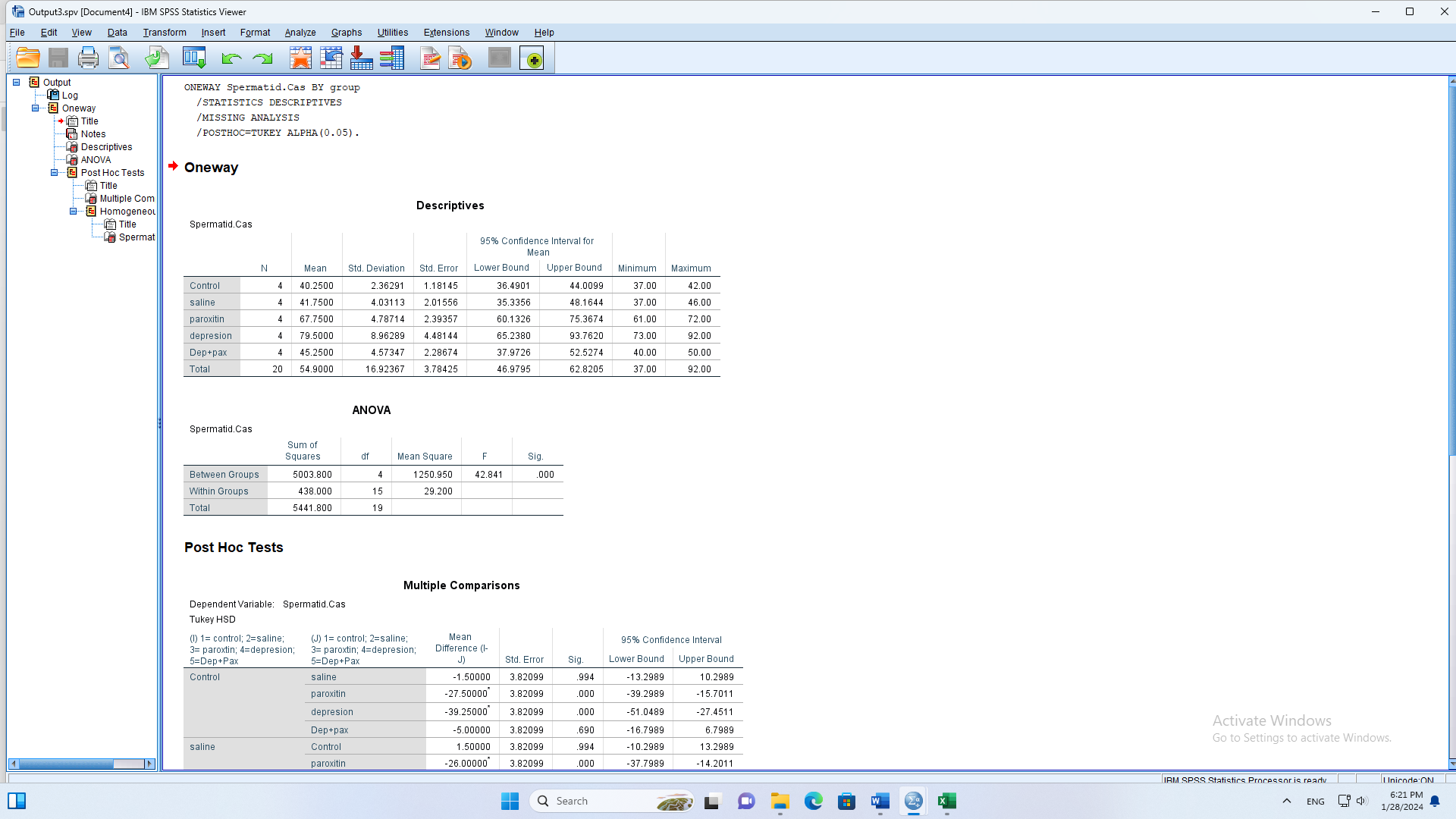

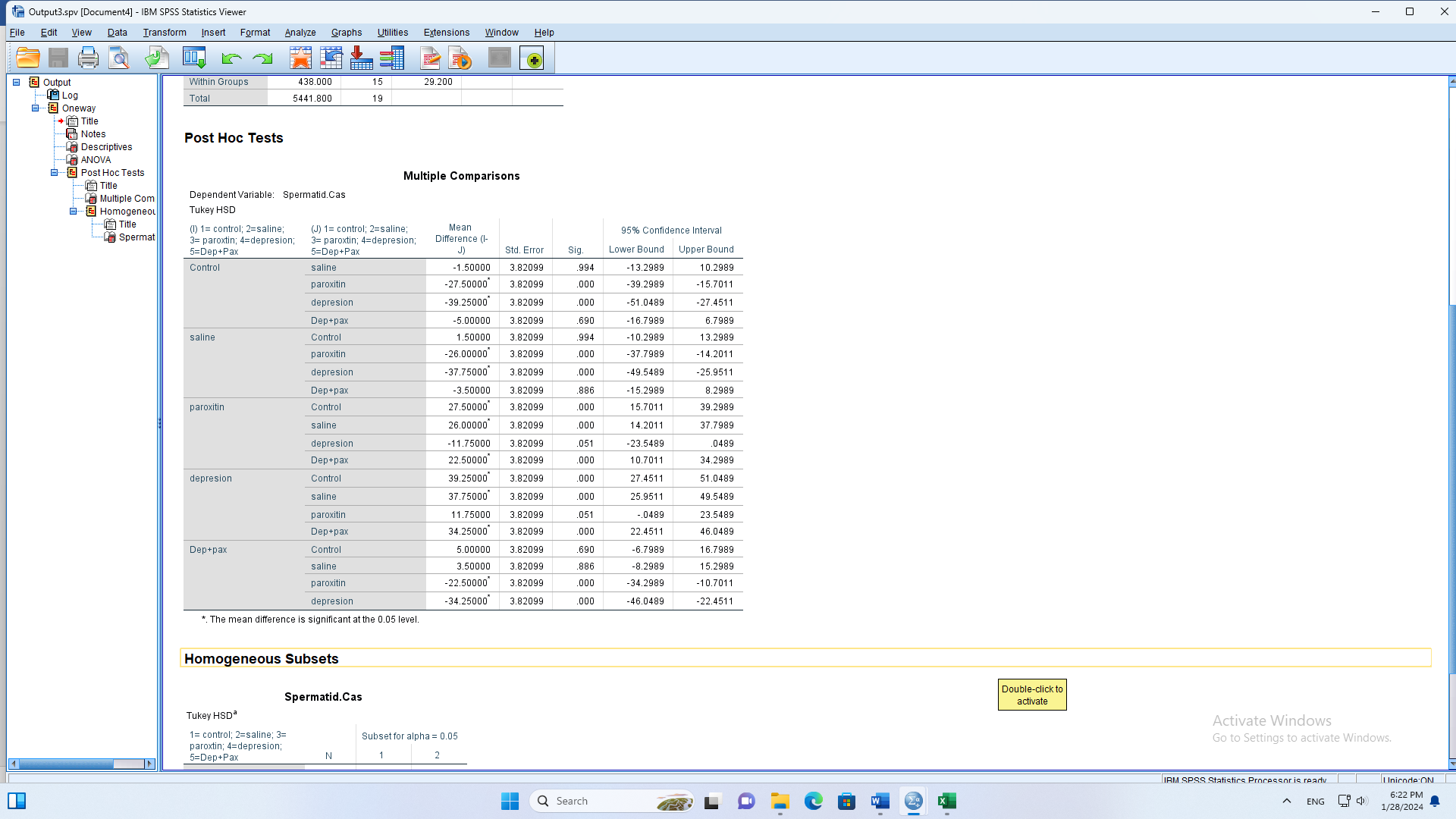


**…………………………………………………………………………………………………….**


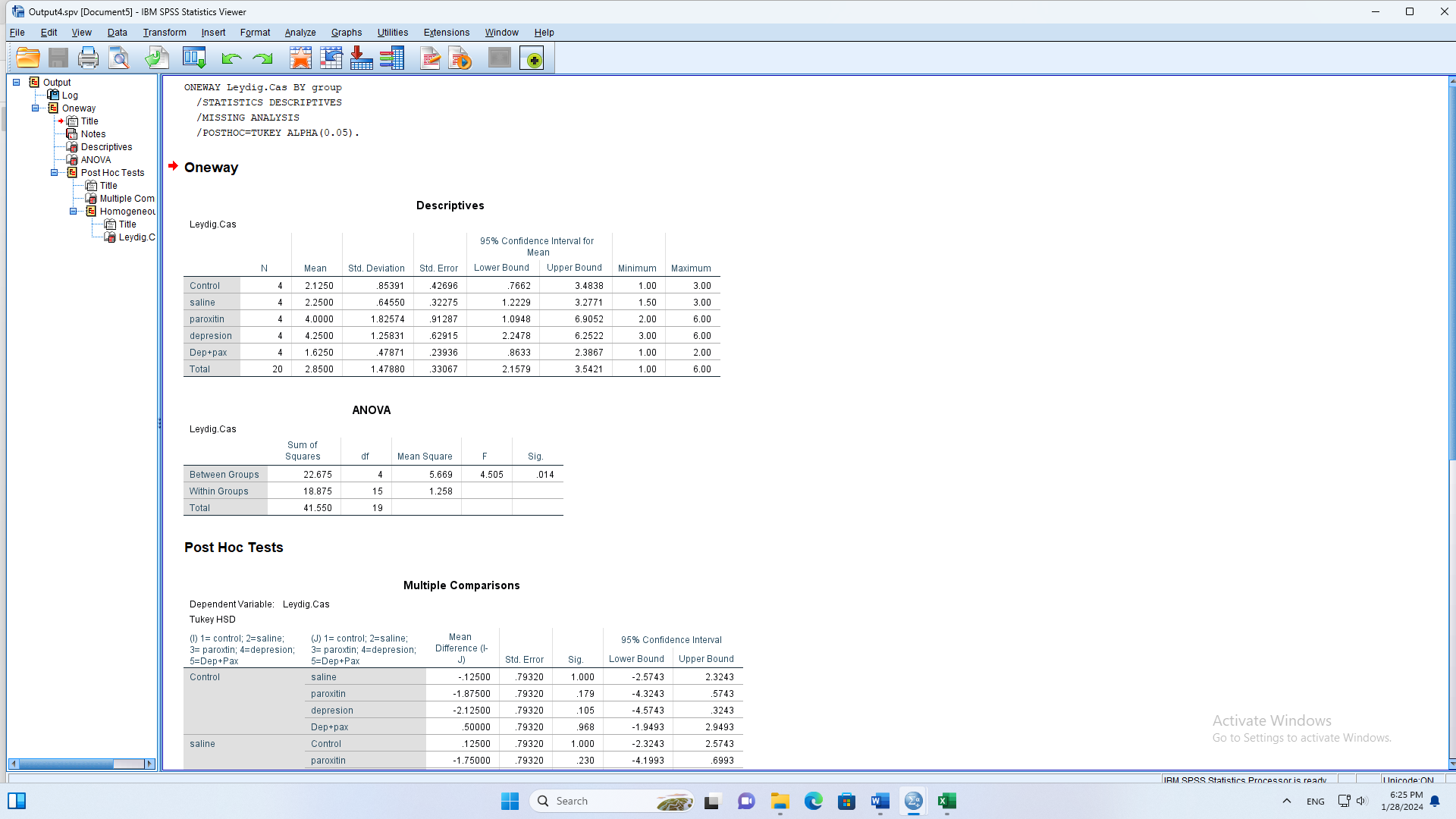

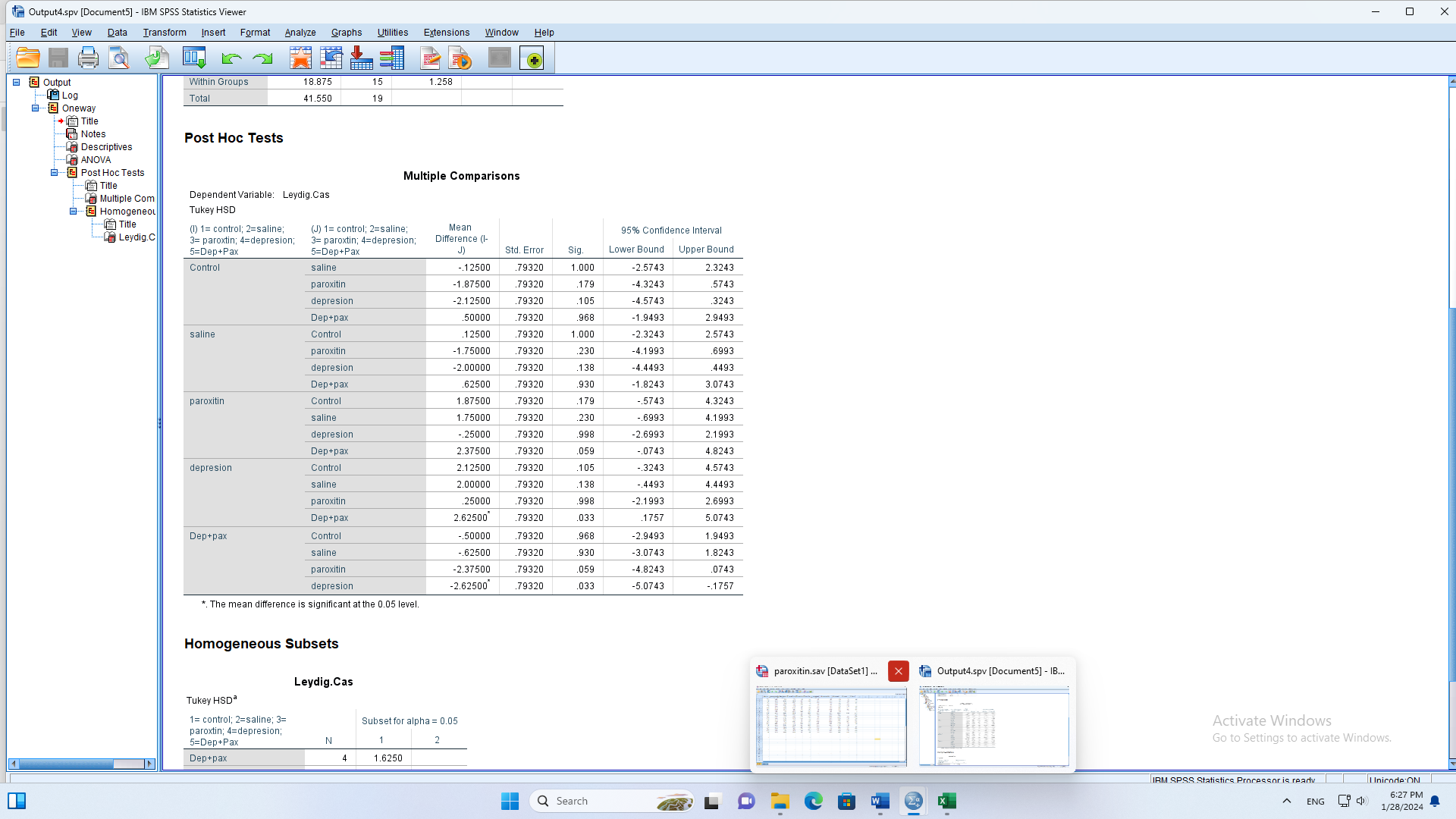


**…………………………………………………………………………………………………….**


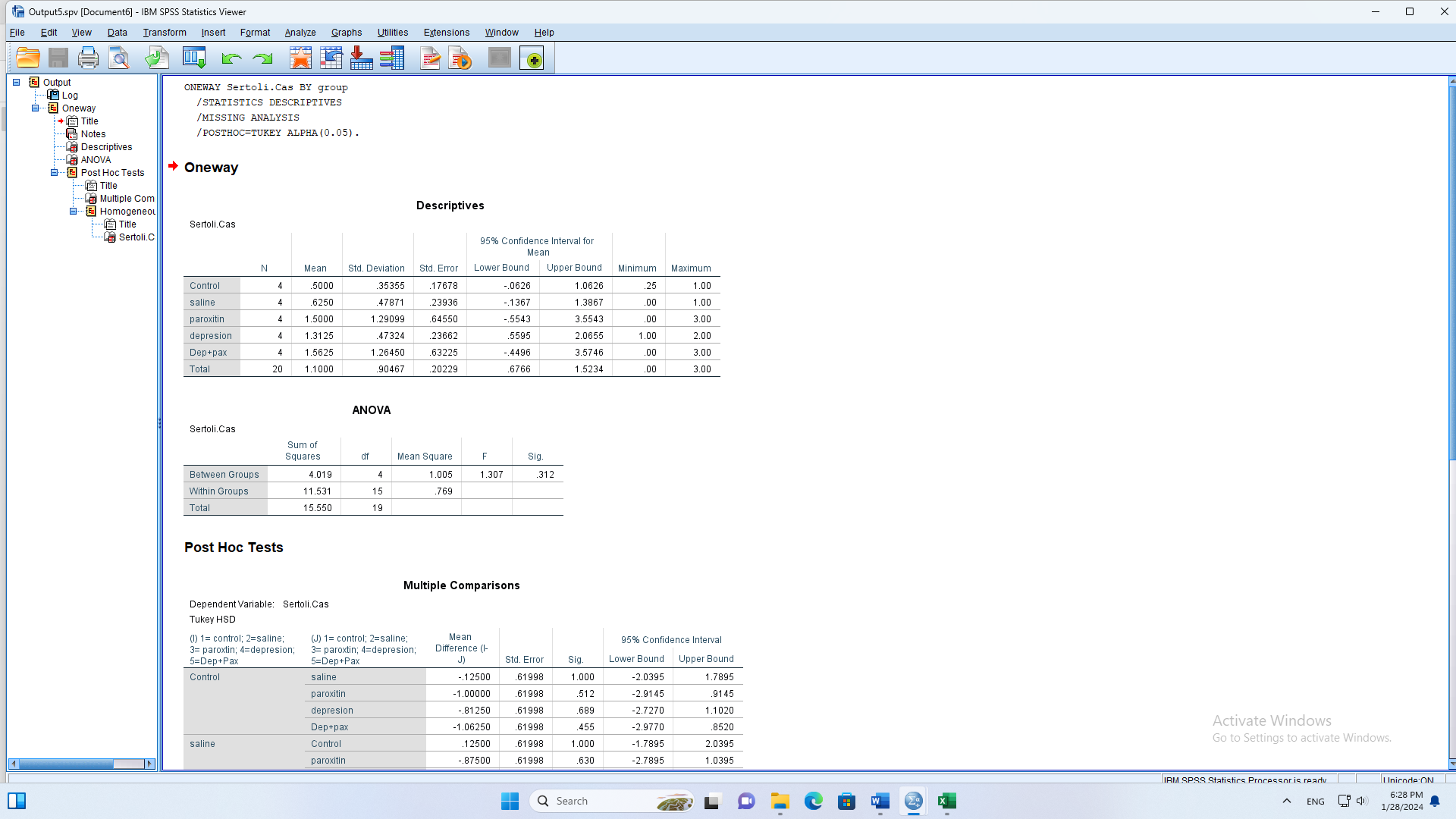

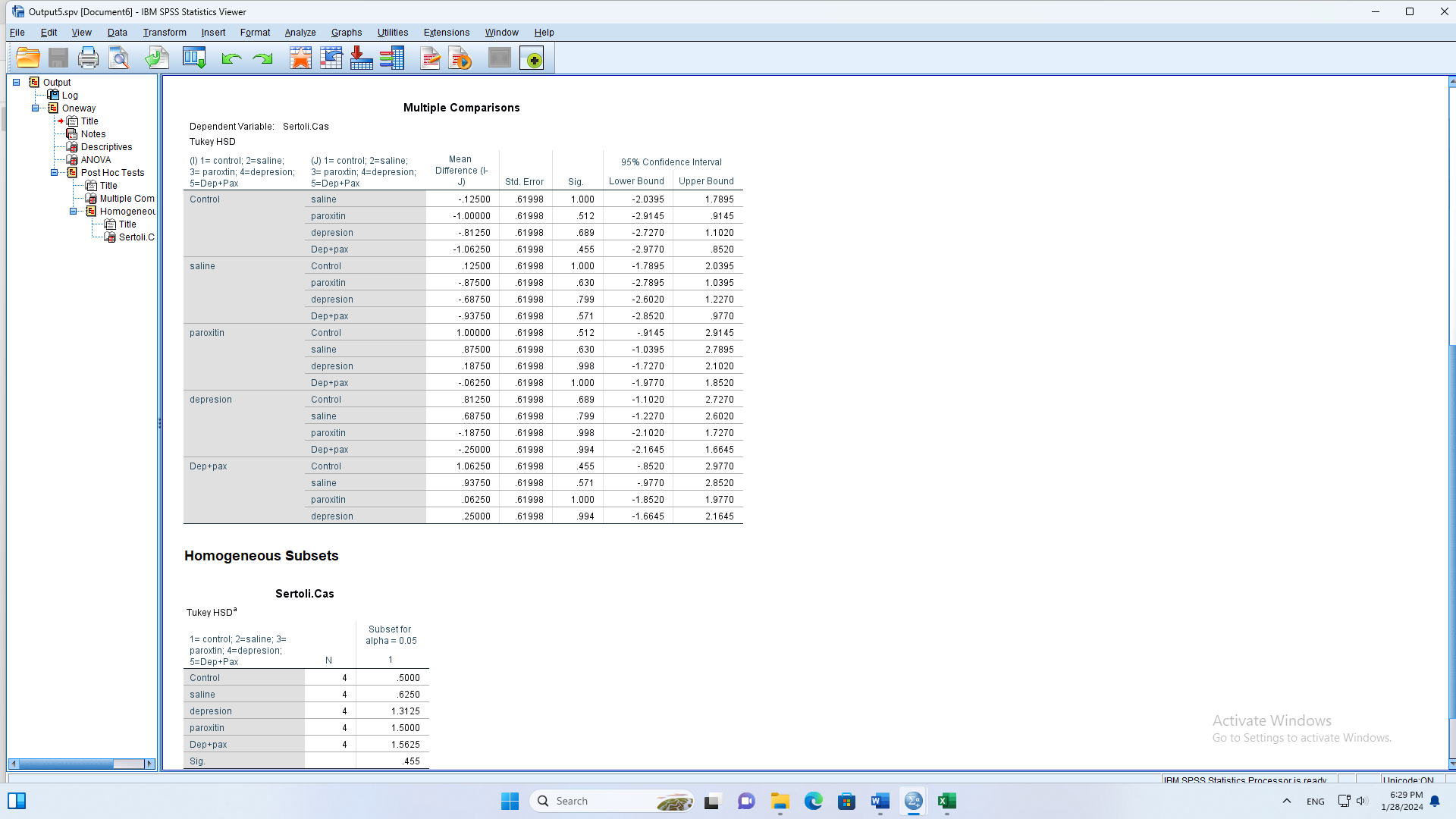

Supplement: S3 File — (DOCX) [file pone.0323480.s003.docx]
