# Supplementary material for: Expression of Concern: Paroxetine treatment in an animal model of depression improves sperm quality
Source: PLoS One. 2025 Apr 24;20(4):e0323480. doi: 10.1371/journal.pone.0323480 (PMC12021233; doi:10.1371/journal.pone.0323480)

Details of Table 3

| Germ cells | Control | Saline | Paroxetine | Depression | Depression  +  Par | P-value |
| --- | --- | --- | --- | --- | --- | --- |
| **Spermatogonia**  Mean ± SEM Mean ± SD | 14 ± 1.8  14 ± 3.7 | 13.75 ± 1.7  13.75 ± 3.3 | 26.5 ± 1.3  26.5 ± 2.6 | 25.5 ± 1.04  25.5 ± 2.1 | 18.75 ± 1.1  18.75 ± 2.2 | Control vs. Paroxetine: P<0.001  Control vs. Depression: P<0.001  Saline vs. Paroxetine: P<0.001  Saline vs. Depression: P<0.001 |
| **Spermatocyte**  Mean ± SEM Mean ± SD | 23.75 ± 2.8  23.75 ± 5.7 | 26.5 ± 2.9  26.5 ± 5.7 | 30.25 ± 1.7  30.25 ± 3.3 | 33.75 ± 1.3  33.75 ± 2.6 | 26.75 ± 2.9  26.75 ± 5.6 | Control vs Depression: P=0.06 |
| **Spermatid**  Mean ± SEM Mean ± SD | 40.25 ± 1.2  40.25 ± 2.4 | 41.75 ± 2.0  41.75 ± 4.0 | 67.75 ± 2.4  67.75 ± 4.8 | 79.5 ± 4.5  79.5 ± 9.0 | 45.25 ± 2.3  45.25 ± 4.6 | Control vs. Paroxetine: P<0.001  Control vs. Depression: P<0.001  Saline vs. Paroxetine: P<0.001  Saline vs. Depression: P<0.001  Paroxetine vs. Depression: P=0.05  Paroxetine vs. Depression+Par: P<0.001  Depression vs. Depression+Par: P<0.001 |
| **Leydig**  Mean ± SEM Mean ± SD | 2.12 ± 0.4  2.12 ± 0.9 | 2.25 ± 0.32  2.25 ± 0.6 | 4.00 ± 0.9  4.00 ± 1.8 | 4.25± 0.6  4.25 ± 1.3 | 1.62 ± 0.2  1.62 ± 0.5 | Paroxetine vs. Depression+Par: P=0.05  Depression vs. Depression+Par: P=0.03 |
| **Sertoli**  Mean ± SEM Mean ± SD | 0.5 ± 0.2  0.5 ± 0.4 | 0.62 ± 0.2  0.62 ± 0.5 | 1.5 ± 0.6  1.5 ± 1.3 | 1.31 ± 0.2  1.31 ± 0.5 | 1.56 ± 0.6  1.56 ± 1.3 | **………………………** |

Figure 3: Immunohistochemical staining of CASPASE-3 in testes cross-sections. Comparison of mean percentage of Caspase-3 -positive cells in germ cells (spermatogonia, spermatocytes, spermatids, Leydig, and Sertoli cells) within groups.


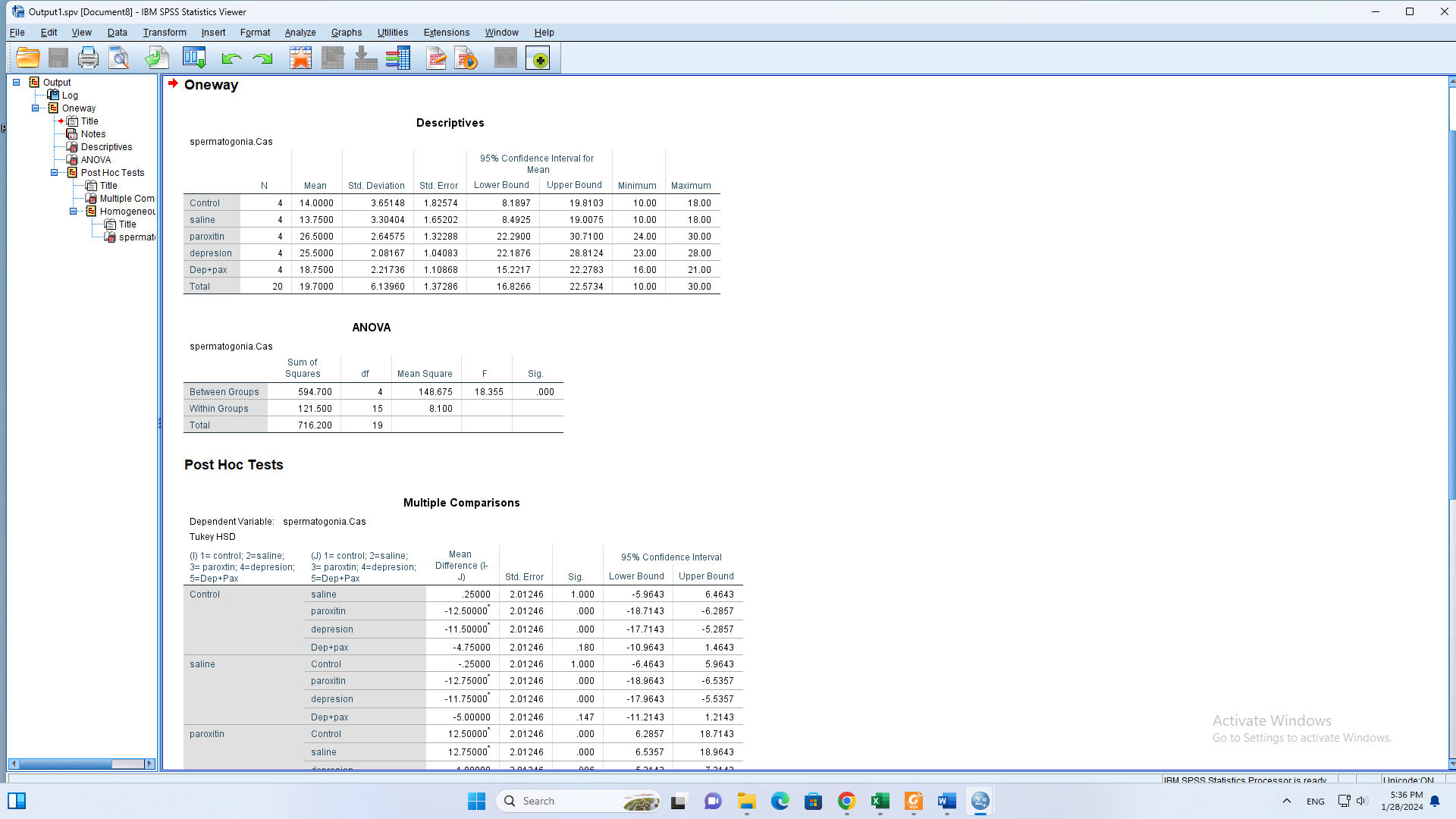

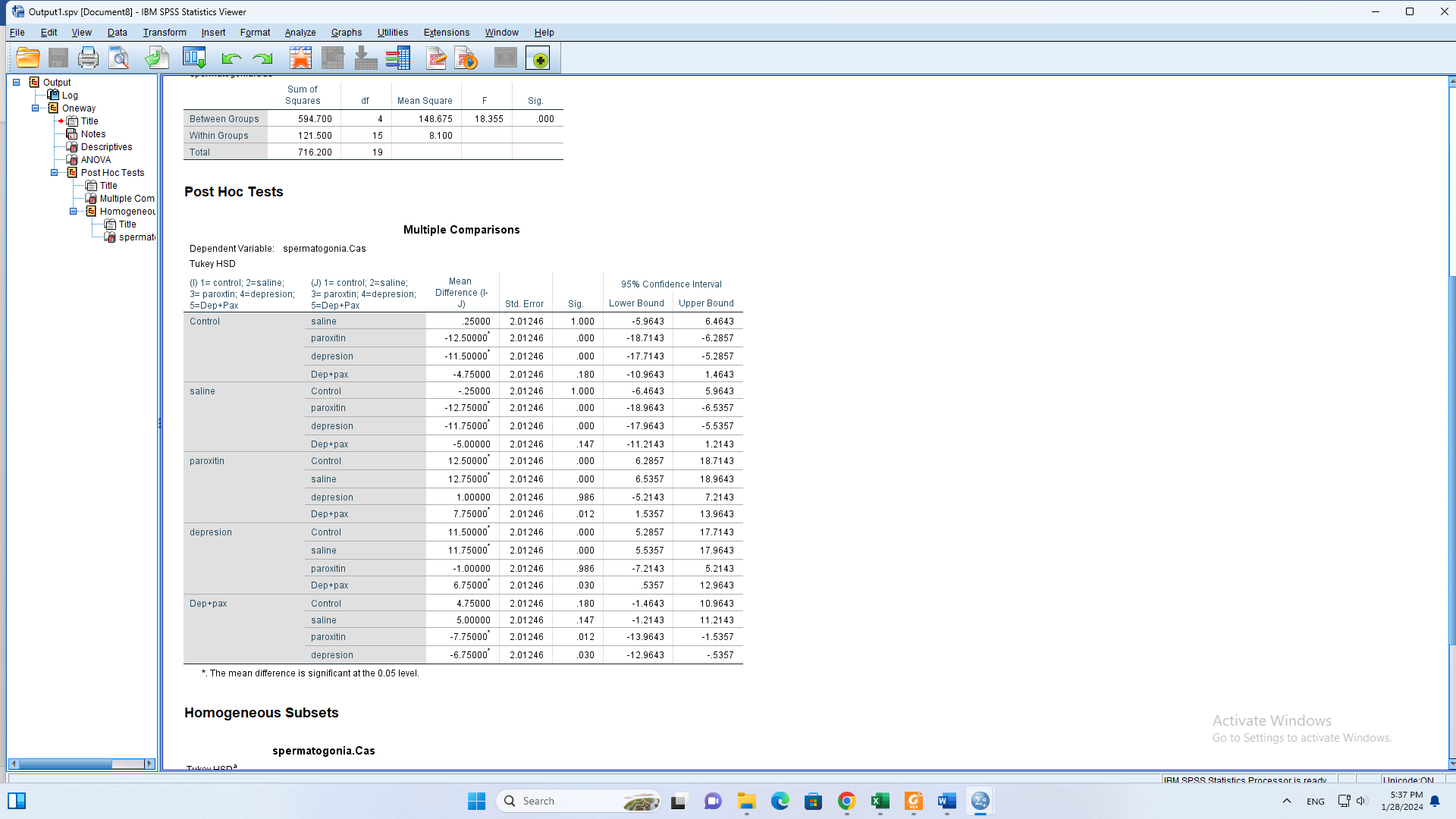


**…………………………………………………………………………………………………………………………………………………….**


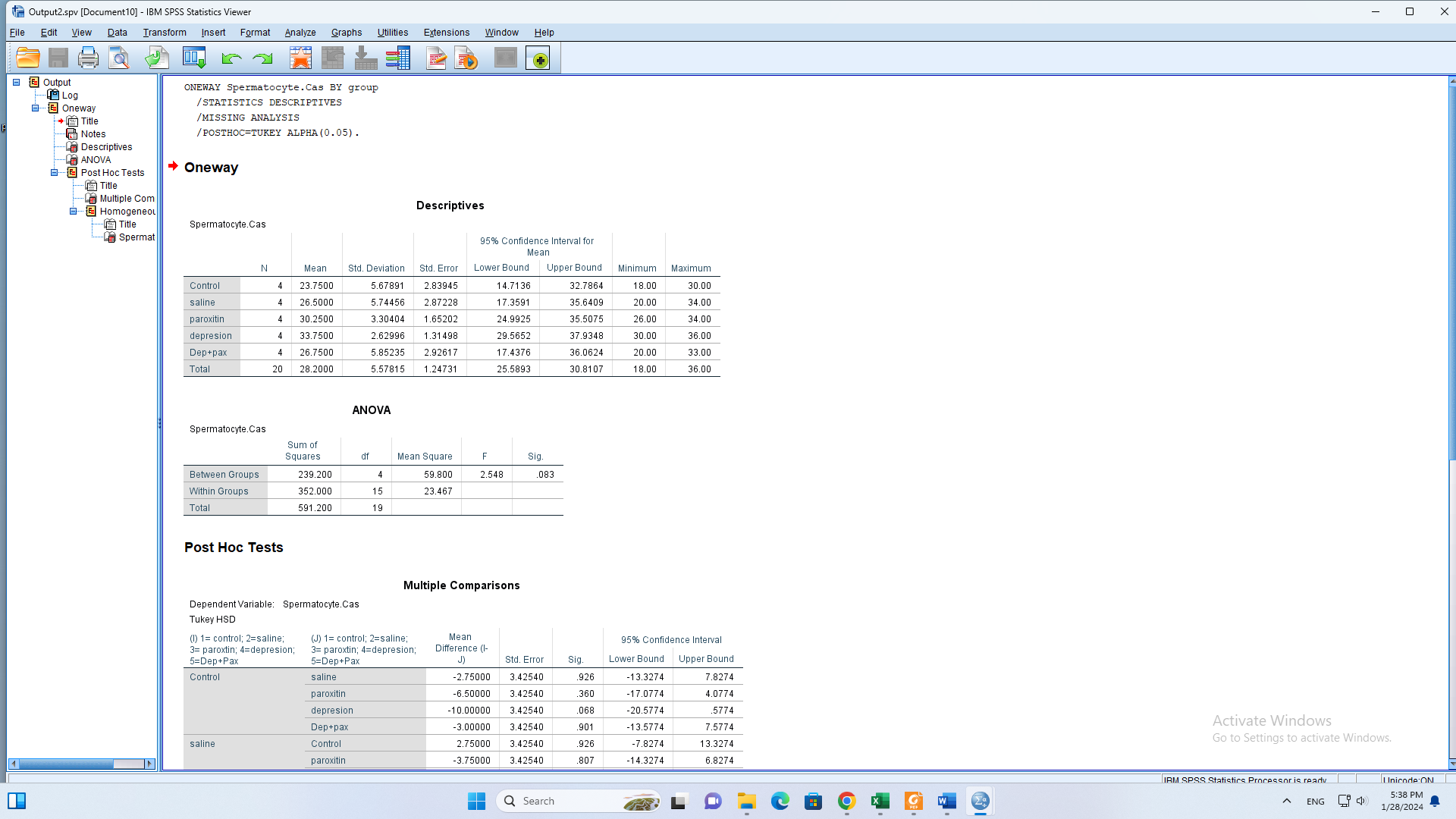

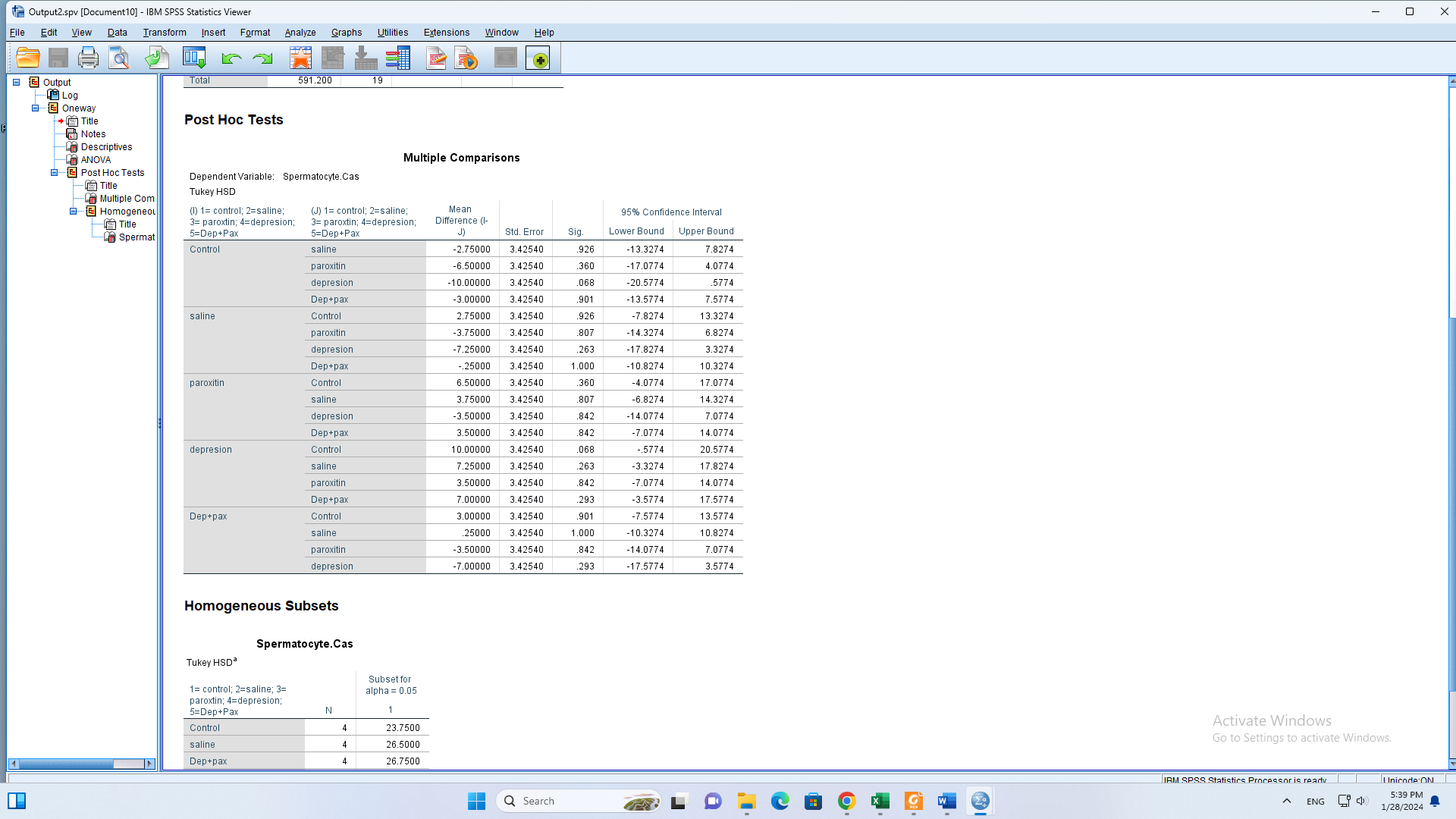


**…………………………………………………………………………………………………………………………………………………………..**


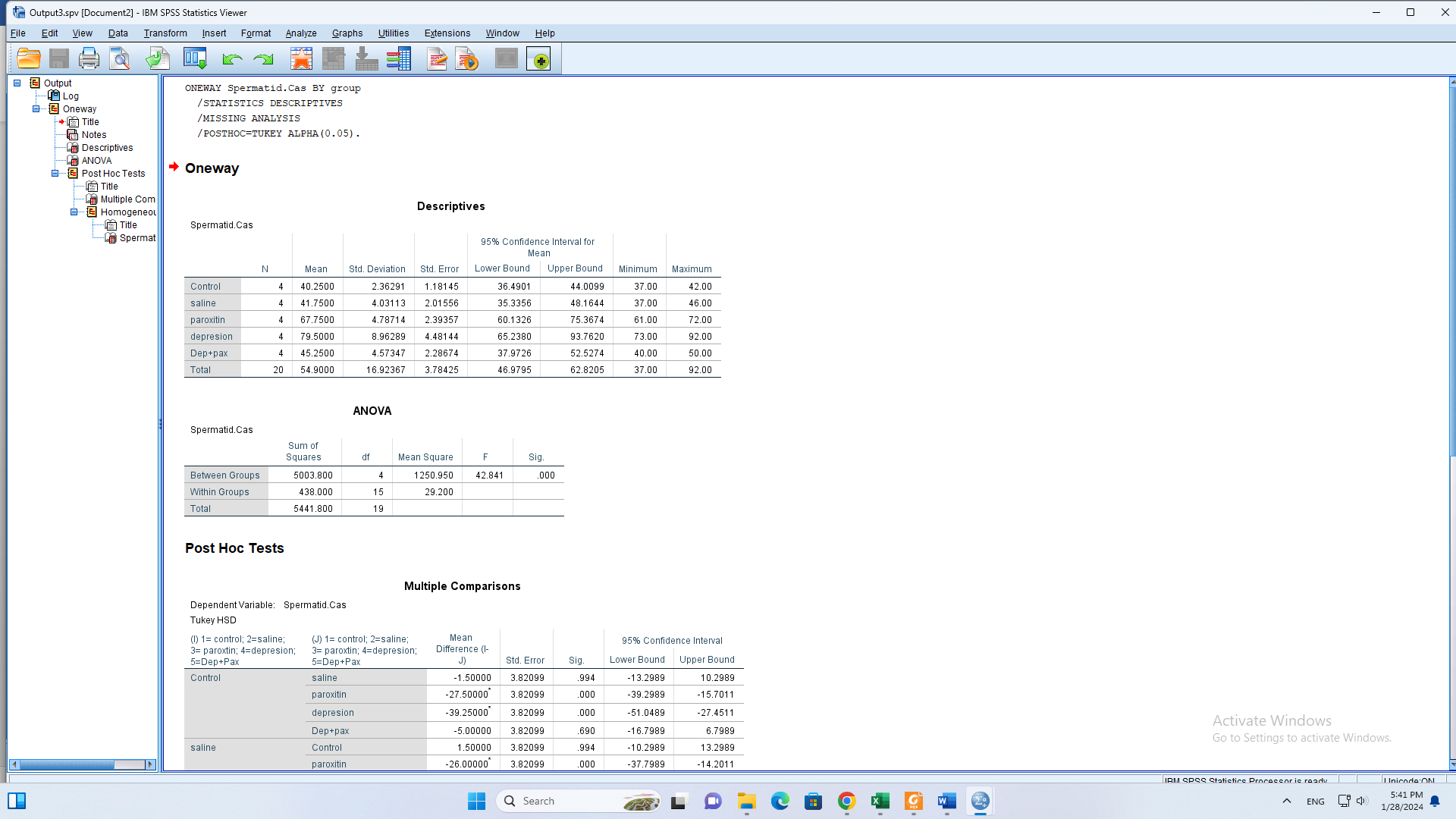

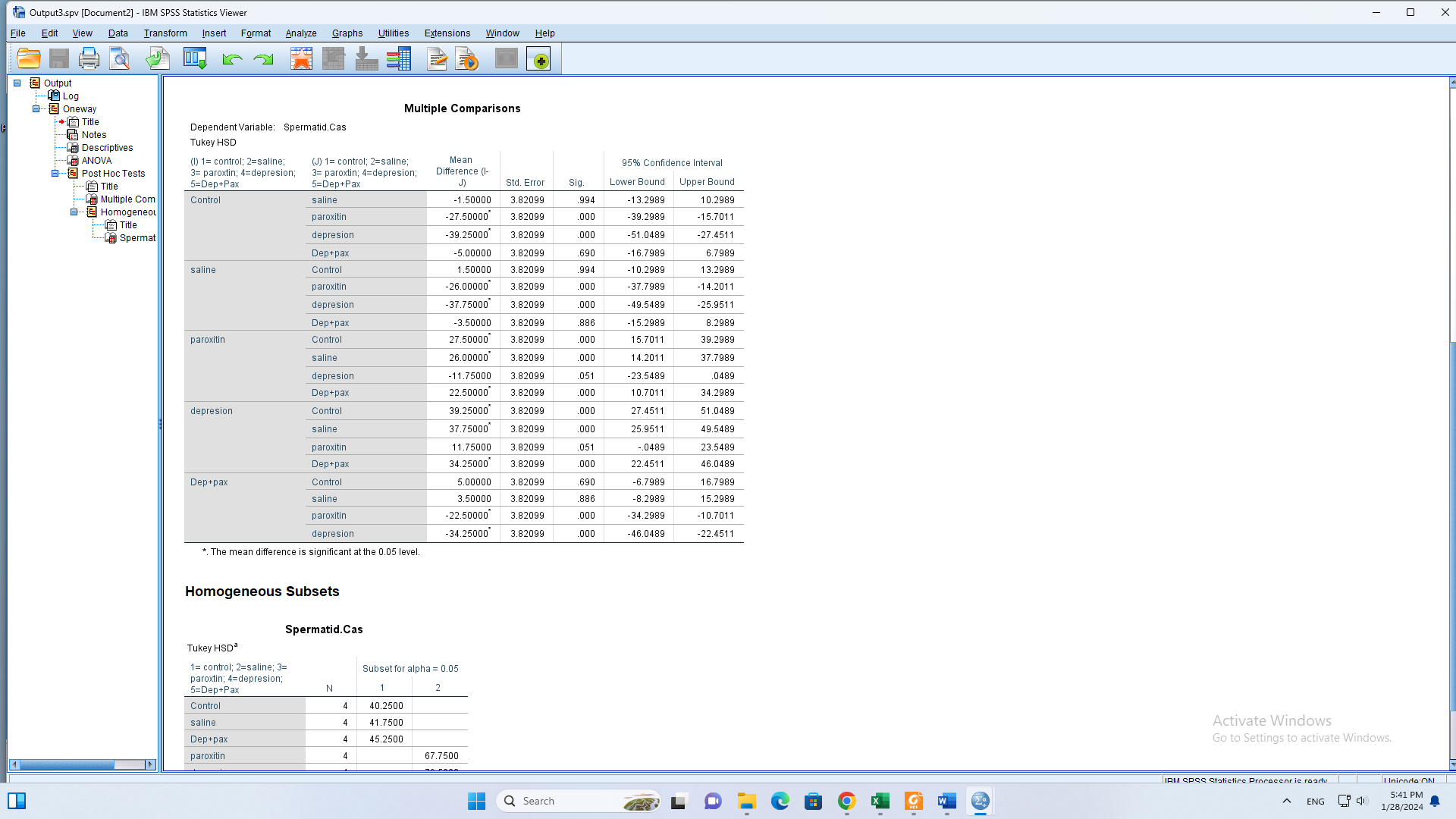


**………………………………………………………………………………….**


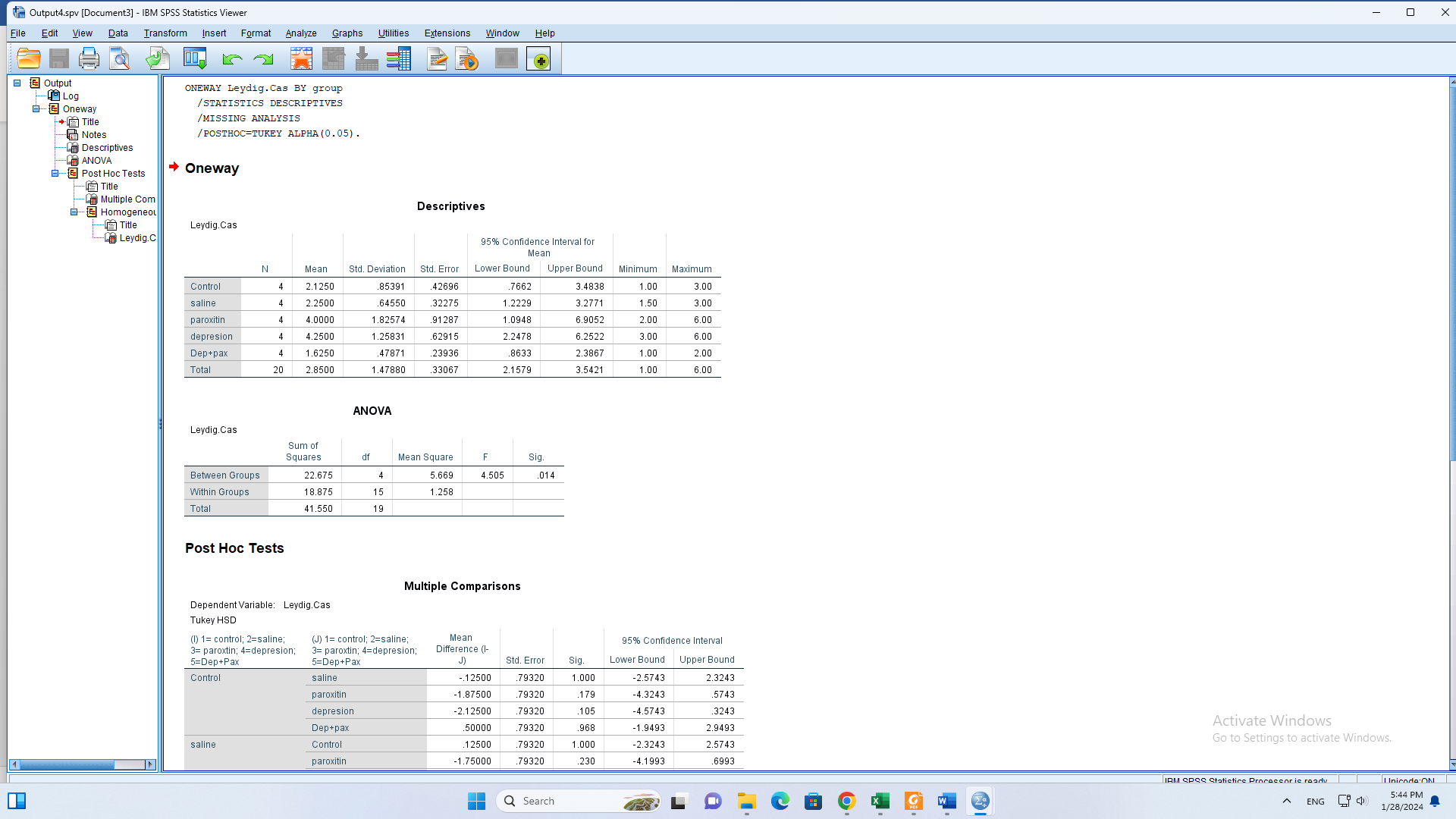

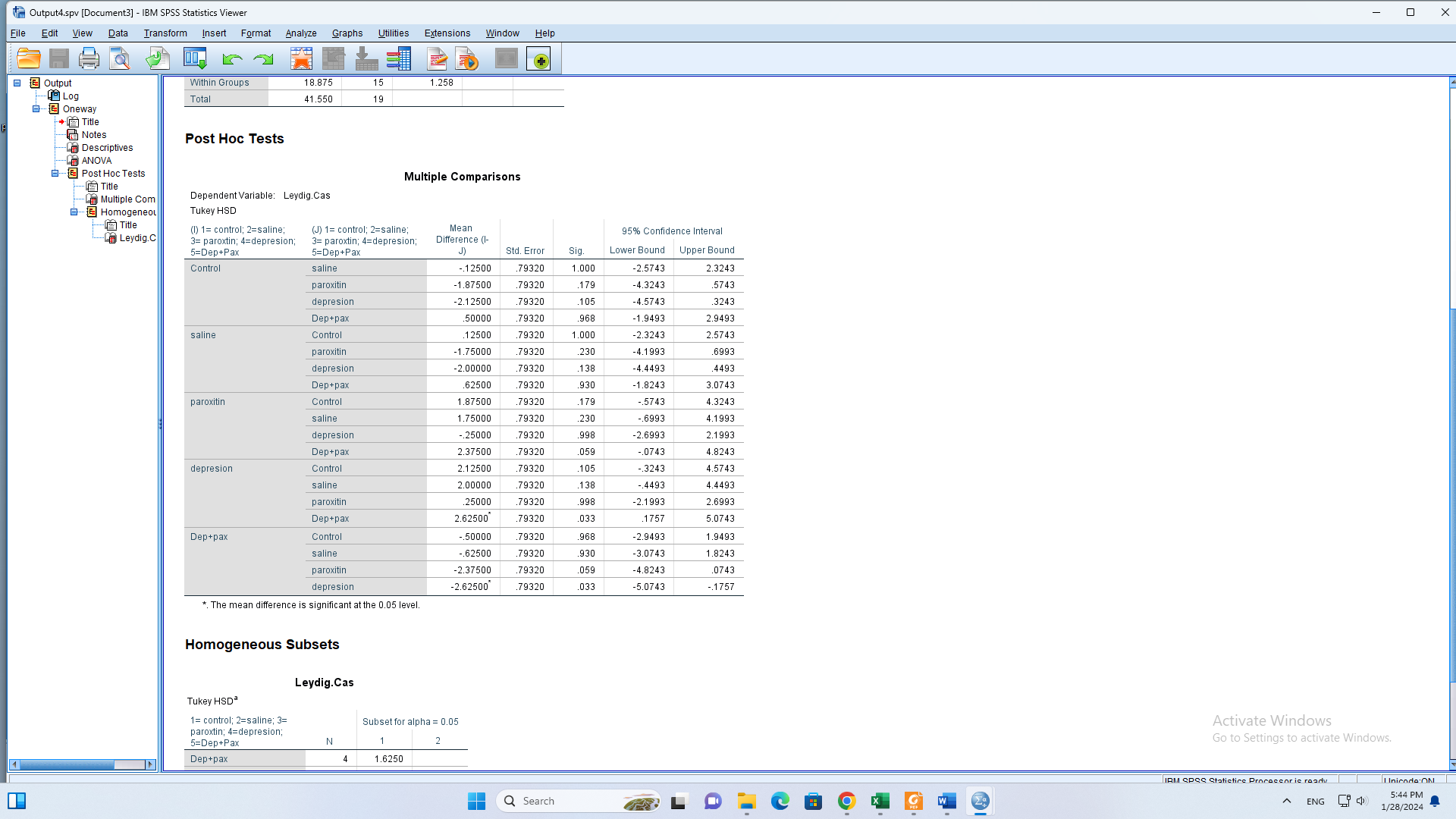


**………………………………………………………………………………….**


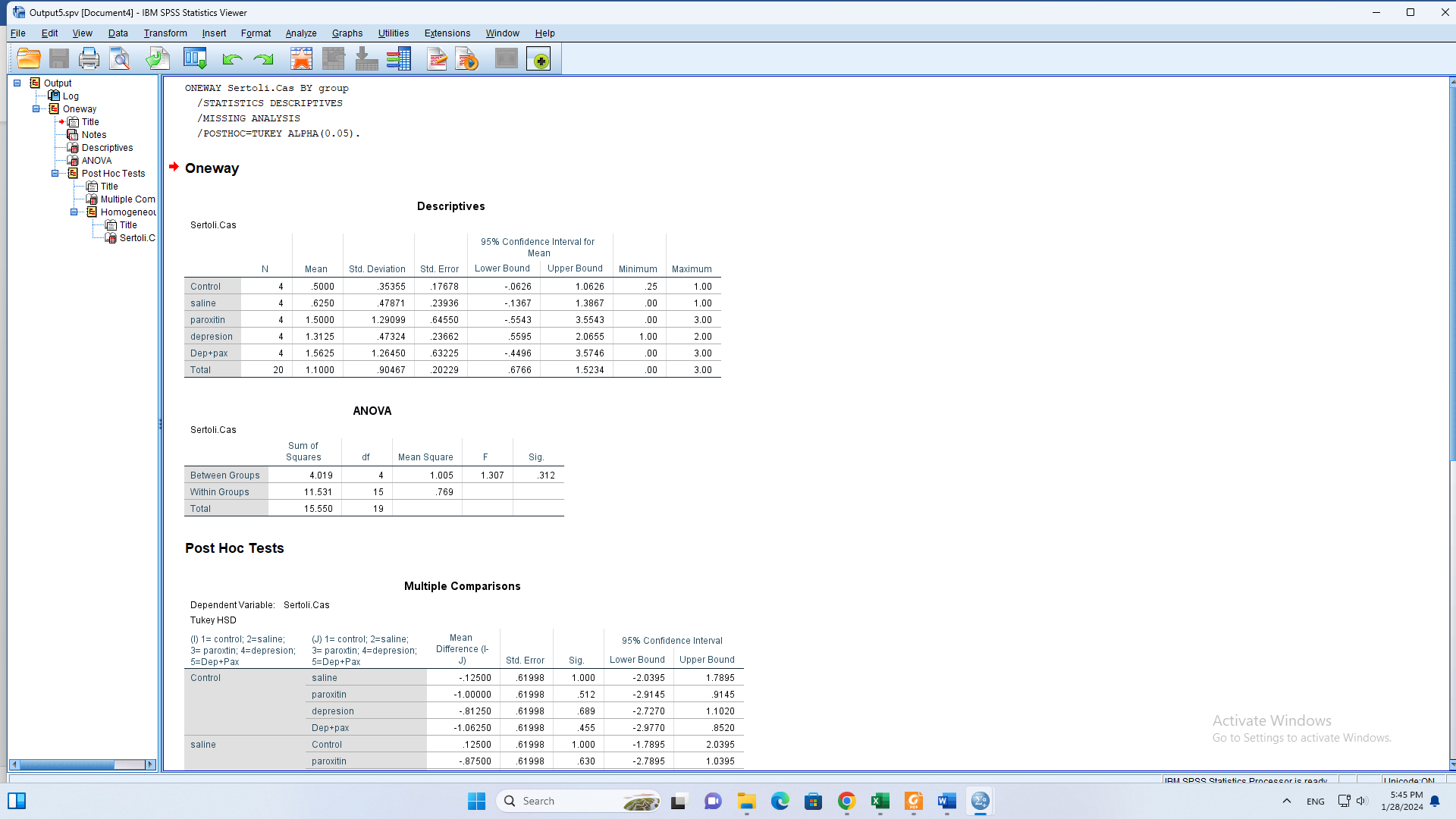

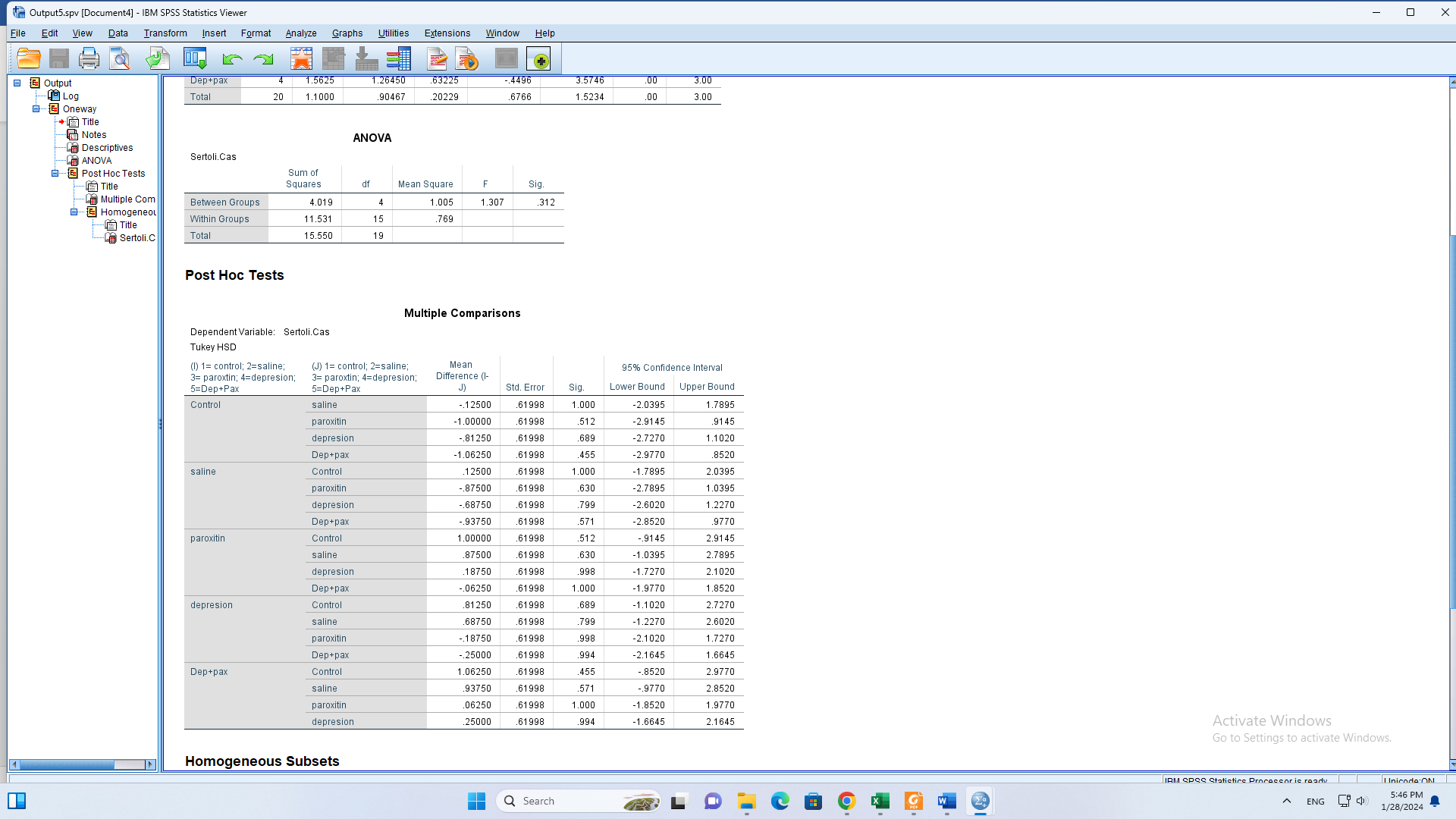

Supplement: S4 File — (DOCX) [file pone.0323480.s004.docx]
